# Industrial Aplication of Catalytic Systems for *n*-Heptane Isomerization

**DOI:** 10.3390/molecules16075916

**Published:** 2011-07-14

**Authors:** Laura Olivia Alemán-Vázquez, José Luis Cano-Domínguez, Enelio Torres-García, José Roberto Villagómez-Ibarra

**Affiliations:** 1 Transformation Processes Program, Mexican Petroleum Institute, Pachuca- Cd Sahagún Road km 7.5, Canacintra Industrial Park, Mineral de la Reforma, Hgo. 42186, Mexico; 2 Transformation Processes Program, Mexican Petroleum Institute, Eje Central Lázaro Cárdenas Norte 152, San Bartolo Atepehuacan, México D. F. 07730, Mexico; 3 Chemical Research Center, Hidalgo State University, Pachuca-Tulancingo Road km 4.5, Mineral de la Reforma, Hgo. 42076, Mexico

**Keywords:** molybdenum sub-oxides, sulfated zirconia, catalyst, *n*-heptane isomerization, X-ray diffraction, Raman spectroscopy

## Abstract

The ideal gasoline must have a high pump octane number, in the 86 to 94 range, and a low environmental impact. Alkanes, as a family, have much lower photochemical reactivities than aromatics or olefins, but only the highly branched alkanes have adequate octane numbers. The purpose of this work is to examine the possibilities of extending the technological alternative of paraffin isomerization to heavier feedstocks (*i.e.*, *n*-heptane) using non-conventional catalytic systems which have been previously proposed in the literature: a Pt/sulfated zirconia catalyst and a molybdenum sub-oxide catalyst. Under the experimental conditions at which these catalysts have been evaluated, the molybdenum sub-oxide catalyst maintains a good activity and selectivity to isomerization after 24 h, while the Pt/sulfated zirconia catalyst shows a higher dimethylpentanes/methylhexanes ratio, probably due to a lower operating temperature, but also a high formation of cracking products, and presents signs of deactivation after 8 h. Though much remains to be done, the performance of these catalysts indicates that there are good perspectives for their industrial application in the isomerization of *n*-heptane and heavier alkanes.

## 1. Introduction

In the formulation of clean gasolines for today’s engines, attention must be given to the technical and environmental quality of these fuels. The ideal gasoline must have a high pump octane number, in the 86 to 94 range, and a low environmental impact. Alkanes, as a family, have much lower photochemical reactivities than aromatics or olefins, but only the highly branched alkanes have adequate octane numbers. Since the alkanes present in the gasoline fraction of crude oils are predominantly linear or monobranched, they must be converted into highly branched isomers through the isomerization and reforming processes. Therefore, the isomerization of butane, pentane and hexane are important technologies for improving the octane number of gasolines in petroleum refining, but this technology has not been applied to *n*C_7_ and heavier alkanes because the formation of cracking products becomes too high as conversion increases. The interest in the production of clean gasolines with high octane numbers has led to the search for new solid catalysts with improved selectivity for *n*C_7_+ isomerization (Tbales 1 and 2).

**Table 1:** Photochemical reactivity for pure components.

Hydrocarbons	Ozone Production (g O_3_/g Hydrocarbon)
**ALKANES**	
*n*-pentane	1.040
*n*-hexane	0.980
*n*-heptane	0.810
*n*-octane	0.610
*n*-nonane	0.540
**BRANCHED ALKANES**	
2-methylbutane	1.380
2-methylhexane	1.080
2-methylheptane	0.960
2,4-dimethylhexane	1.500
2,2,4-trimethylpentane	1.600
**ALKENES**	
1-pentene	6.220
1-octene	5.290
3-octene	5.290
4-methyl-1-pentene	4.420
**AROMATICS**	
Toluene	2.730
Ethylbenzene	2.700
*m*-xylene	7.380
*p*-xylene	7.380

Compounds of groups V, VI and VII transition metals have been widely used as catalysts for the isomerization of *n*-alkanes. The carbides of tungsten and molybdenum are characterized by their high thermal stability and surface reactivity in heterogeneous catalysis. It has been shown that the oxidation of these carbides leads to active and selective materials for the isomerization of alkanes [1,2,3]. Molybdenum sub-oxides have been prepared by direct reduction of MoO_3_ with a carbon-rich source at temperatures around 623 K [1,2,3]. The reduction of MoO_3_, during which different molybdenum sub-oxide phases are formed, is the crucial step in the formation of active catalysts for the isomerization of alkanes and has been widely studied [4,5,6,7]. Ledoux *et al*. have suggested that the active phase for alkanes isomerization in this catalyst is a carbon-containing molybdenum sub-oxide, or molybdenum oxycarbide (MoO_x_C_y_), where carbon atoms fill oxygen vacancies in the course of the catalyst activation, and that the isomerization reaction proceeds through a bond-shift mechanism via a metallocyclobutane intermediate. However, studies carried out by Iglesia *et al.* [8] on an analogous tungsten catalyst have shown that the isomerization reaction proceeds through the conventional bifunctional mechanism, with dehydrogenation-hydrogenation on metallic sites (WC_x_) and C–C bond rearrangement on acid sites (WO_x_).

**Table 2:** Octane numbers of heptane isomers.

C_7_ ALKANES	OCTANE NUMBERS		
	Research	Motor	Pump
2,2,3-trimethyl butane	112.10	101.30	106.70
2,2-dimethyl pentane	92.80	95.60	94.20
2,4-dimethyl pentane	83.10	83.80	83.45
3,3-dimethyl pentane	80.80	86.60	83.70
2,3-dimethyl pentane	91.10	88.50	89.80
2-methyl hexane	42.40	46.40	44.40
3-methyl hexane	52.00	55.80	53.90
3-ethyl pentane	65.00	69.30	67.15
*n*-heptane	0.00	0.00	0.00

The oxidation state of Mo in the molybdenum suboxides catalyst affects its acidity [9] and reduction with hydrogen has substantial effects on the surface acidity and catalytic activity of this catalyst in the isomerization of heptanes [10].

On the other hand, some solid superacid catalysts are known to catalyze the skeletal isomerization of hydrocarbons at low temperatures, but these catalysts tend to deactivate quickly. In particular, sulfated zirconia (SZ) is a very strong solid acid that is able to catalyze the isomerization of *n*-hexane at room temperature, even in the absence of metal sites, but undergoes a very fast deactivation. Several postulates have been offered to explain the rapid catalyst deactivation, including loss of sulfur, change in sulfur oxidation state, transformation of the crystalline structure and deposition of hydrocarbon fragments. The sulfur content could influence the catalyst´s crystalline structure and activity [11,12]; when amorphous zirconium hydroxide is treated with sulfate groups, the crystallization process is retarded [13], as sulfate groups relieve some surface energy, thereby stabilizing the tetragonal phase. It has been observed that the addition of platinum increases the stability of SZ catalysts [14,15,16,17] and the presence of hydrogen during the reaction is essential to maintain their catalytic activity. The role of platinum in the presence of hydrogen is still a controversial issue; some authors suggest that strong protonic acidity is generated via dissociation and spillover of hydrogen species and the conversion of hydrogen atoms into hydrides, accelerating the desorption of the carbenium intermediates [17,18].

Alkane isomerization on Pt/SZ proceeds via chain transfer pathways, with a chain initiation step involving loss of hydrogen from alkanes, a carbenium ion isomerization step and carbenium ions propagation by hydrogen transfer from a neutral molecule to a carbocation, in which an isomerized carbocation is removed from the surface and replaced by a new carbocation. Carbocations desorption is slow and limited by the rate of hydrogen transfer, leading to long surface residence times and a high probability that cracking via β-scission reactions occurs, particularly for *n*-heptane and higher alkanes, for which oligomerization is not required for the β-scission reaction to proceed. The surface isomerization step is quasi-equilibrated and the surface is almost saturated with carbocations at steady-state [17]. These characteristics of the Pt/SZ catalysts indicate that if the desorption rate can be increased, the cracking selectivity could be reduced while maintaining a good approach to equilibrium in the reaction product isomers distribution. It has been shown that the addition of small amounts of adamantane (0.8 wt %) to *n*-heptane increases isomerization rates by a factor of 3 and decreases cracking rates, resulting in a five-fold improvement in the isomerization/cracking selectivity ratio [17].

Acid strength is probably the most important factor in isomerization activity and the importance of Bronsted acid sites has been demonstrated. The surface structure of Pt/SZ has been studied in order to understand the nature of the active sites, and various structures have been proposed of the involved species such as:

(a) The strong acidity of sulfated zirconia has been attributed to the electron-withdrawing anion groups, which lead to coordinatively unsaturated and electron defficient metal centers that behave as strong Lewis acid sites. Water vapor titrates such Lewis sites and converts them to Bronsted acids with very reactive protons [17].
(b) Arata and Hino [19] proposed that water adsorption generates the Lewis and Bronsted acid sites responsible for the catalytic activity, which was confirmed by IR-spectroscopy using pyridine as the test molecule.
(c) Yamaguchi [20], using IR-spectroscopy, showed that superacid centers are Lewis sites associated to the metallic cation. Acid strength of these sites is intensified by the inductive effect of the electrons of the double bond in the S=O structure.
(d) Babou *et al.* [21] considered the acid sites of the sulfated zirconia as H_2_SO_4_ molecules supported on the zirconia surface, which can be reversibly hydrated. At high dehydration conditions an adsorbed SO_3_ species with a high Lewis acidity is obtained. In an intermediate hydration stage, the presence of H_3_O^+^ and HSO_4_^−^ species promote a high protonic Bronsted acid strength. This reversible effect of water is important for catalytic applications because it modifies the system acidity.

Our research is focused on *n-*heptane and higher alkanes isomerization with non-commercial catalysts for improving the octane number of gasolines. The present work describes the synthesis, characterization and evaluation of both the Pt/SZ and the molybdenum sub-oxide catalytic systems for *n*-heptane isomerization, two of the most promising new catalysts proposed for paraffins isomerization.

## 2. Results and Discussion

The specific surface area of the Pt/SZ catalyst was 306 m^2^/g before calcination and after calcination it decreased to 129 m^2^/g. This decrease can be related to densification of the ZrO_2_ structure, loss of micro- and mesopores and the formation of the monoclinic phase [22]. It is known that transformation from tetragonal to monoclinic zirconia is accompanied not only by a loss in surface area, but also by a corresponding increase in the average crystallite size [23,24]. By contrast, the specific surface area of the pre-activated molybdenum sub-oxide catalyst after 4 h of reduction with a mixture of H_2_/*n*C_7_ increases from less than 20 m^2^/g to 53 m^2^/g.

**Figure 1:**
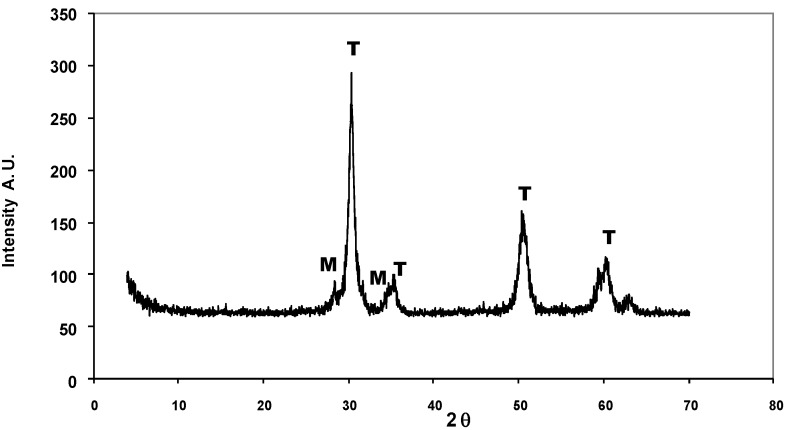
X-ray difraction of the Pt/SZ catalyst after calcination at 873 K.

X-ray difraction of the Pt/SZ catalyst before calcination shows only broad bands in the 18–40° and 40–70° ranges in angular displacement (2θ), indicative of the small size of the crystallites or of amorphous behavior. Highly crystalline materials were obtained after calcination at 873 K (Figure 1). Both monoclinic (M: 2θ ≈ 28°) and metastable tetragonal (T: 2θ ≈ 30°) phases are detected; the latter is predominant [25]. These results suggest that when amorphous zirconium hydroxide is impregnated with sulfate groups, these groups stabilize the metastable tetragonal phase of ZrO_2_ and delay the sintering process [25]. The tetragonal phase has been reported to be the active phase for isomerization in SZ catalysts.

**Figure 2:**
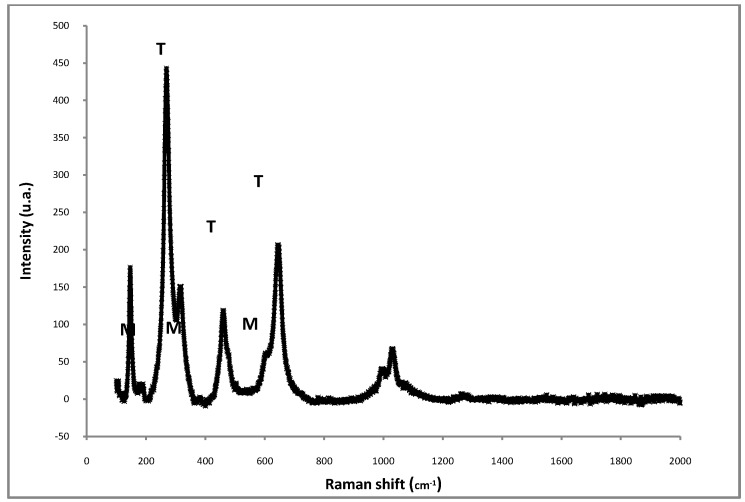
Raman spectra of Pt/SZ catalyst after calcination at 873 K.

Raman spectroscopy was used to study the structure of sulfated zirconia at a molecular length scale. Figure 2 shows the Raman spectrum of the SO*_x_*-ZrO_2_ sample. The tetragonal and monoclinic ZrO_2_ polymorphs presented Raman bands in the 100–760 cm^−1^ region. In general, the typical tetragonal phase of ZrO_2_ can be identified from the bands at 317, 455 and 640 cm^−1^, whereas the bands at 223, 560 and 622 cm^−1^ are assigned to the monoclinic phase [22]. The bands at 1000 and 1031 cm^−1^ can be attributed to S–O modes of the sulfate groups (adsorbed species of SO_3_ and H_2_SO_4_). The band located at 1070 cm^−1^ can also be attributed to S–O stretching modes, but in polynucleate sulfate groups. 

**Figure 3:**
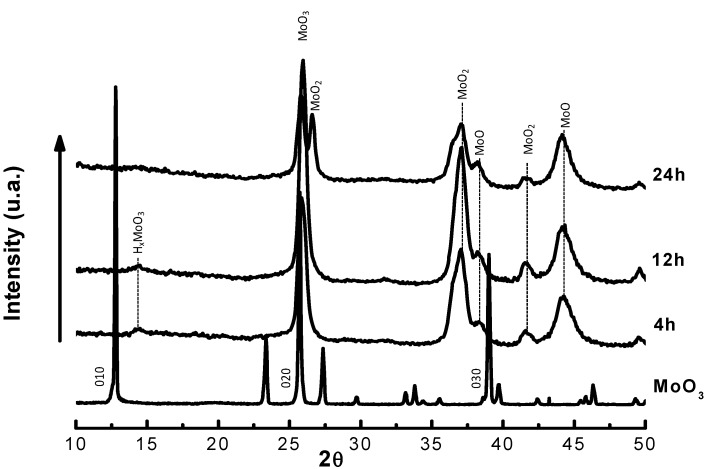
XRD patterns of I) orthorhombic MoO_3_ (JCPDS data file no. 05-0508) and molybdenum sub-oxides obtained at 4, 12 and 24 h under flow of H_2_/*n*-C_7_ at 643 K.

XRD patterns of the molybdenum sub-oxides obtained by reduction with H_2_/*n*C_7_ at 643 K and 18.5 bar for 4, 12 and 24 h are presented in Figure 3. The XRD pattern of the starting orthorhombic MoO_3_ (JCPDS data file no. 05-0508) is included for better understanding. Besides MoO_3_, new phases were detected after the reduction process. One of the phases is unequivocally related to the monoclinic MoO_2_ (JCPDS data file no. 32-671). The low intensity peak at *d_hkl_* = 6.1 Å is associated to the most intense reflection line (10-1) of residues of a hydrogen molybdenum bronze H_x_MoO_3_ (JCPDS data file no. 41-0060). Reflections with interplanar distances at 2.34 and 2.04 Å are associated to the formation of a molybdenum sub-oxide with a cubic crystal structure, having an experimental cubic parameter a = 4.08 Å, previously identified as MoO [26,27,28].

**Figure 4:**
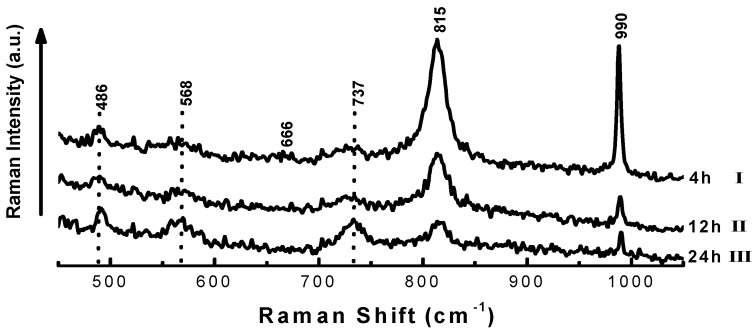
Raman spectra of the MoO_3_ samples after treatment with H_2_/*n*C_7_ at 643 K for 4 h (I), 12 h (II) and 24 h (III).

In general, the Raman spectra of the MoO_3_ samples after treatment with H_2_/*n*-C_7_ at 643 K for 4, 12 and 24 h show significant changes in the intensity of the Raman bands, see Figure 4. These changes are associated with the degree of crystallinity of the samples and the progressively lower oxygen/metal ratio. Raman bands at 666, 815 and 990 cm^−1^ correspond to orthorhombic MoO_3_, while bands at 486, 568 and 737 cm^−1^ are characteristic of monoclinic MoO_2_ obtained during the experiments, which is in agreement with our XRD results. These results suggest that the significant decrease in intensity and resolution of the bands at 815 and 990 cm^−1^ during reduction is associated to the preferential electrophilic attack of the H_2_ on the terminal *π* M=O bonds along the {0k0} planes, with the formation of oxygen vacancies and partial reduction of the Mo^+6^. These vacancies increase the density of surface sites on the basal planes and lead to the formation of crystallographic shear planes during the reduction process. These segments are thought to be catalytically active for chemical reactions, as Mo cations on them are coordinately unsaturated.

In this work, we carried out the isomerization tests of the Pt/SZ and molybdenum sub-oxides catalysts under operating conditions suggested in previous studies. The conversion and products distribution of *n*-heptane isomerization with the Pt/SZ catalyst at 473 K and at 2, 4, 6 and 8 h are shown in Table 3. This catalyst showed a maximum conversion around 53% and a fair amount of isomerization products, with methylhexanes and dimethylpentanes as the main products. The dimethylpentanes/methylhexanes ratio was relatively high (0.374–0.423), probably due to the low reaction temperature, but still far from the thermodynamic equilibrium ratio of 1.8. However, a high formation of cracking products (C_1_–C_6_) was observed for this catalyst under these experimental conditions, resulting in relatively low selectivities to isomerization (70%–78%). The *n*-heptane conversion decreased from 6 to 8 h on stream. The causes of their deactivation are still under discussion. The deactivation has been attributed to deposition of polymerized hydrocarbons on the catalyst, causing the coverage of the acid centers by carbonaceous deposits [29], to reduction of Zr^4+^ to Zr^3+^ on the surface of the catalysts during the reaction or to the transformation of tetragonal to monoclinic zirconia on the surface of the catalyst [30].

The conversion and products distribution of *n*-heptane isomerization with the molybdenum sub-oxide catalyst at 643 K and at 2 to 24 h are shown in Table 4. The conversion of *n*-heptane increased during this period and reached a high of 78% at 24 h. This slow development of the catalyst’s activity suggests that the active phase is formed progressively by the reduction of the MoO_3_ and stabilizes after 12 to 24 h on stream. No deactivation was observed after 24 h on stream. The dimethylpentanes/methylhexanes ratio started around 0.2 and increased to a high of 0.325 after 24 h; this ratio was lower than the one for the Pt/SZ catalyst, probably due to the higher reaction temperature (643 K), for which the thermodynamic equilibrium ratio is close to 1. The formation of cracking products was moderate up to 24 h on stream, increasing somewhat after 12 h.

**Figure 5:**
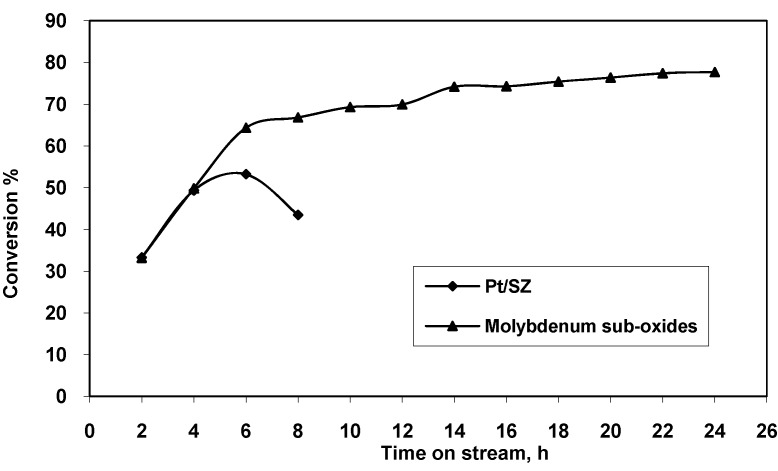
Conversion of *n*-heptane for Pt/SZ and molybdenum sub-oxide catalysts at 18.5 bar.

The conversion of *n*-heptane on the molybdenum sub-oxides and Pt/SZ catalysts are shown in Figure 5.

**Table 3:** Isomerization of *n-*heptane over Pt/SZ catalyst (473 K, 18.5 bar).

Time on stream (h)	2	4	6	8
Conversion (wt %)	33.33	49.35	53.23	43.47
Selectivity to isomerization (wt %)	73.96	70.49	69.96	77.57
Selectivity to cracking (wt %)	7.58	25.21	26.38	19.14
Branched isomers (wt %)	24.48	34.54	36.97	33.48
Dimethylpentanes	6.50	10.02	10.66	9.15
Methylhexanes	17.37	23.69	25.46	23.55
3-ethylpentane	0.61	0.83	0.86	0.78
Dimethylpentanes/ Methylhexanes	0.37	0.42	0.41	0.39
Cracked products (wt %)				
C1–C4	1.30	5.02	5.70	3.22
C5–C6	1.20	7.32	8.23	5.04

**Table 4:** Isomerization of *n*-heptane over molybdenum sub-oxides catalyst (643 K, 18.5 bar).

Time on stream (h)	2	4	6	8	10	12	14	16	18	20	22	24
Conversion (wt %)	33.21	49.84	64.36	66.85	69.31	70.24	74.21	74.43	75.43	76.39	77.45	78.01
Selectivity to isomeriz. (wt %)	89.94	92.82	92.11	92.12	90.68	92.33	89.79	89.43	87.93	86.44	85.34	87.45
Selectivity to cracking (wt%)	7.85	4.59	7.31	7.62	9.07	7.47	9.77	10.17	11.64	12.81	14.16	11.99
Branched isomers (wt %)	29.66	45.94	58.87	61.15	62.41	64.40	66.17	66.10	65.86	65.57	65.63	67.74
Dimethylpentanes	4.67	7.44	10.72	11.59	12.36	12.98	14.31	14.44	14.70	14.89	15.29	16.07
Methylhexanes	24.11	37.01	46.14	47.50	47.91	49.21	49.61	49.41	48.92	48.50	48.16	49.40
3-ethylpentane	0.89	1.49	2.01	2.06	2.14	2.22	2.25	2.24	2.24	2.18	2.17	2.26
DimethylC_5_/ MethylC_6_	0.194	0.201	0.232	0.244	0.258	0.264	0.288	0.292	0.300	0.307	0.317	0.325
Cracked products (wt %)												
C_1_–C_4_	1.16	1.23	1.74	1.91	2.78	2.16	3.21	3.58	4.55	5.32	6.32	4.96
C_5_–C_6_	1.43	1.04	2.93	3.15	3.46	3.05	3.99	3.94	4.17	4.40	4.57	4.33

## 3. Experimental

Two isomerization catalysts were synthesized: (i) a Pt/sulfated zirconia (SZ) catalyst, prepared by the precipitation method from zirconium oxychloride; and (ii) a molybdenum sub-oxide catalyst, obtained by reduction of MoO_3_.

### 2.1. Sulfated Zirconia (SZ) Catalyst Preparation

High-surface-area ZrO_2–x_(OH)_2x_ (320 m^2^/g) was prepared by hydrolysis of ZrOCl_2_·8H_2_O (Aldrich, 98% purity) by addition of an NH_4_OH solution (J.T. Baker, 28 wt %) under agitation, until a pH value of 10 was reached. The precipitate was filtered and washed repeatedly by re-dispersion with NH_4_OH solution (pH = 10) until elimination of Cl^−^, and then dried at 383 K for 24 h. Subsequently, ZrO_2–x_(OH)_2x_ was sulfated by impregnation during 4 h with a 0.07 M solution of H_2_SO_4_ (for a theoretical sulfur concentration of 3 wt %). The sulfated material was vacuum filtered and dried at 383 K for 15 h and then immersed in an aqueous solution of H_2_PtCl_6_ (Aldrich, 8 wt %) to obtain 0.3 wt % of platinum in the catalyst and stirred during 30 min. The material was dried at 383 K for 15 h and calcined at 873 K for 2 h. The sulfur content in the Pt/SZ catalyst before calcination was determined as 2.95 wt % and after calcination as 1.18 wt %. The reduction in the sulfur content after calcination may be related to a high proportion of the sulfate ions being on the surface of the catalyst.

### 2.2. Molybdenum Sub-Oxides Catalyst Preparation

A 1 g MoO_3_ sample (Fermont, 98 wt % purity) was placed in a continuous flow stainless steel reactor (0.9 cm in diameter) and the temperature was raised to 643 K under hydrogen flow of 100 mL/h. The pressure was kept at 18.5 bar. Afterwards, *n*-heptane (Aldrich, 99 wt % purity) was also fed to the reactor at a rate of 5 mL/h during 4 h using an Eldex metering pump. The hydrogen flow was controlled using a Brooks 5850E mass flow controller.

### 2.3. Characterization

The specific surface areas of the samples were determined by the BET method using a Micromeritics ASAP-2401 system. The MoO_3_, the molybdenum sub-oxides catalyst and the Pt/SZ catalyst were characterized by X-ray diffraction using a Siemens diffractometer (Model D5000) with Cu K_α_ radiation and a Ni filter. The operating conditions were 30 kV and 20 mA in the angular range 4–70° in 2θ. Crystalline phase identification based on XRD patterns was aided by the ICDD-PDF-2 database. The Raman spectra of MoO_3_, of the molybdenum sub-oxides catalyst and of the Pt/SZ catalyst were obtained in air at room temperature with a double monochromator Raman spectrometer (SPEX Mod. 1403) using an Ar^+^ ion laser which delivered 10 mW of incident radiation. The excitation line of the laser was 514.5 nm. The Raman signal was detected with a photomultiplier and a standard photon counting system. Total sulfur in the Pt/SZ catalyst was determined by the ASTM D1552 analytical method.

### 2.4. Catalysts Evaluation

#### 2.4.1. Isomerization reaction with the Pt/SZ catalyst

A 1 g sample of this catalyst was placed in a continuous flow stainless steel reactor (0.9 cm in diameter) and the temperature was raised to 473 K under hydrogen flow at a pressure of 18.5 bar. Hydrogen flow (100 mL/h) was controlled using a Brooks 5850E mass flow controller. Then *n*-heptane (Aldrich, 99 wt % purity) was fed to the reactor at a rate of 5 mL/h using an Eldex metering pump, at the pressure above indicated.

#### 2.4.2. Isomerization reaction with the molybdenum sub-oxides catalyst

The isomerization reaction was carried out under the same flows and conditions used for the molybdenum sub-oxide preparation (643 K, 18.5 bar). The activity and selectivity of both catalysts for the isomerization of *n*-heptane were evaluated at different reaction times with on-line analysis of the reaction products using an Agilent 6890 gas chromatograph equipped with the PIANO software. The conversion and selectivity were calculated using the following equations: 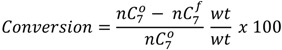


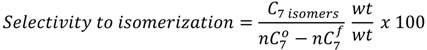

where 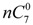 and 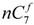 are the concentration of 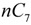 at the beginning of the test and at each sampling time respectively.

## 4. Conclusions

The Pt/SZ catalyst (1.18 wt % sulfur, 0.3 wt % Pt) showed a better dimethylpentanes/methylhexanes ratio (0.374–0.423), a maximum *n*-heptane conversion of 53% and relatively low selectivities to isomerization (70%–78%), due to a high formation of cracking products. This catalyst presented signs of deactivation after 8 h on stream.

The molybdenum sub-oxide catalyst showed a good activity, reaching a maximum *n*-heptane conversion of 78% after 24 h on stream. No deactivation was observed for this catalyst. The selectivity to isomerization was relatively high (85%–93%) and the formation of cracking products was moderate up to 24 h on stream. However, the dimethylpentanes/methylhexanes ratio was relatively low (0.325 at 24 h).

Though much remains to be done, these results indicate that these catalysts have good perspectives for their industrial application of isomerization to *n*-heptane and heavier paraffins.
